# Prognostic value of the neutrophil-to-lymphocyte ratio in acute ischemic stroke patients treated with intravenous thrombolysis: a systematic review and meta-analysis

**DOI:** 10.1186/s12883-021-02222-8

**Published:** 2021-05-11

**Authors:** Chengbing Wang, Qian Zhang, Mingwei Ji, Jing Mang, Zhongxin Xu

**Affiliations:** 1 Department of Neurology, China-Japan Union Hospital of Jilin University, Xiantai Street NO.126, Jilin Changchun, China; 2 Department of Cardiology, China-Japan Union Hospital of Jilin University, Jilin Changchun, China

**Keywords:** Acute ischemic stroke, Neutrophil-to-lymphocyte ratio, Intravenous thrombolysis, Hemorrhagic transformation, Functional outcome, Meta-analysis

## Abstract

**Background:**

The relationship between the neutrophil-to-lymphocyte ratio (NLR) and poor prognostics in acute ischemic stroke (AIS) patients who receive intravenous thrombolysis (IVT) remains controversial. The purpose of this systematic review and meta-analysis was to evaluate the association between the NLR and poor prognosis after IVT. Furthermore, we aimed to determine whether the NLR at admission or post-IVT plays a role in AIS patients who received IVT.

**Methods:**

The PubMed, Embase, Web of Science and China National Knowledge Infrastructure databases were searched for relevant articles until October 7, 2020. Cohort and case-control studies were included if they were related to the NLR in AIS patients treated with IVT. Odds ratios (ORs) and 95 % confidence intervals (95 % CIs) were pooled to estimate the relationship between NLR and poor prognosis after IVT. A random effects model was used to calculate the pooled data.

**Results:**

Twelve studies, including 3641 patients, met the predefined inclusion criteria. Higher NLRs were associated with an increased risk of hemorrhagic transformation (HT) (OR = 1.33, 95 % CI = 1.14–1.56, *P* < 0.001) and a poor 3-month functional outcome (OR = 1.64, 95 % CI = 1.38–1.94, *P* < 0.001) in AIS patients who received IVT. Subgroup analysis suggested that the NLR at admission rather than post-IVT was associated with a higher risk of HT (OR = 1.33, 95 % CI = 1.01–1.75, *P* = 0.039). There was no statistically significant difference between higher NLRs and 3-month mortality (OR = 1.14, 95 % CI = 0.97–1.35, *P* = 0.120).

**Conclusions:**

A high NLR can predict HT and poor 3-month functional outcomes in AIS patients who receive IVT. The NLR at admission rather than the post-IVT NLR was an independent risk factor for an increased risk of HT after IVT.

**Supplementary Information:**

The online version contains supplementary material available at 10.1186/s12883-021-02222-8.

## Background

Stroke is a leading cause of death and acquired disability worldwide, which brings huge psychological and economic burdens to patients and society [1]. Ischemic stroke accounts for approximately 70 % of all strokes [1]. Intravenous recombinant tissue plasminogen activator (rt-PA) is an effective treatment for AIS patients within 4.5 h after onset [2]. However, there are still some patients who receive IVT with poor functional outcome, and HT is the most common and serious complication of IVT [3].

Inflammation is involved in early brain injury and tissue repair after stroke at a later stage [4]. The NLR is a readily available, repeatable new inflammatory biomarker. Neutrophils are rapidly recruited to the ischemic area after AIS [5]. Neutrophils release reactive oxygen species (ROS) and a variety of inflammatory mediators [6]. This aggravated brain injury and increased the risk of HT. Some types of lymphocytes play an important role in the protective mechanism of ischemic brain tissue [7]. However, systemic immunosuppression and the stress response decrease not only the number but also the activity of lymphocytes [8]. Previous meta-analyses have shown that the baseline NLR was associated with HT and poor functional outcome in AIS patients [9, 10]. However, most of these reviews did not mention whether patients receiving IVT therapy were included. Recent clinical trials have shown that the NLR was associated with HT and poor outcome after IVT. However, the timing of blood sampling varied in these clinical trials. In some studies, the blood sample was collected at admission, whereas other studies suggested that the NLR is a dynamic variable and that the NLR after IVT is more predictive of poor prognosis than the NLR at admission [11, 12]. The purpose of this study was to provide a better understanding of the relationship between the NLR and poor prognosis in AIS patients receiving IVT.

## Methods

### Search strategy

This systematic review and meta-analysis was reported in accordance with the Preferred Reporting Items for Systematic Reviews and Meta-Analyses (PRISMA) Statement [13]. We searched the PubMed, Embase, Web of Science and China National Knowledge Infrastructure databases for relevant studies until October 7, 2020. No language restrictions were imposed. We used the following keywords and combined their synonyms: “neutrophil to lymphocyte ratio”, “intravenous thrombolysis”, and “tissue plasminogen activator”. Taking PubMed as an example, the complete search strategy was as follows: ((neutrophil to lymphocyte ratio[Title/Abstract]) OR (NLR[Title/Abstract])) AND ((“Tissue Plasminogen Activator”[Mesh]) OR (tissue plasminogen activator[Title/Abstract]) OR (alteplase[Title/Abstract]) OR (rtPA[Title/Abstract]) OR (intravenous thrombolysis[Title/Abstract])). The reference lists of included studies were also screened to supplement the search.

### Study selection

Studies that met the following inclusion criteria were selected: (1) the subjects were AIS patients who received IVT; (2) the NLR was measured at admission or after IVT; (3) at least one of the following outcomes was reported: HT, poor functional outcome (modified Rankin Scale ≥ 3), or mortality; and (4) the study design was a cohort or a case-control study. Animal studies, repeatedly published or data overlapping studies, reviews, case reports, conference abstracts, letters, and unrelated articles were excluded. Two independent reviewers screened the titles and abstracts and reviewed the full texts of the studies that met the inclusion criteria. All disagreements were resolved by consulting another reviewer.

### Data extraction and quality assessment

Two reviewers extracted and cross-checked the data independently, and all disagreements were resolved through discussion. The following data were collected: name of the first author, publication year, country, number of patients, sex, age, blood sample collection time, best cutoff value of the NLR, baseline NIHSS score, odds ratio (OR) and 95 % confidence interval (CI) of HT, poor functional outcome and mortality. Multivariate regression analysis was used to calculate the pooled OR if both univariate and multivariate regression analyses were available in the studies. We used the Newcastle-Ottawa Scale (NOS) to evaluate the quality of cohort studies and case-control studies. The study with the highest quality scored 9 points and which scored 7 or more was regarded as high quality [14].

### Statistical analysis

A random effects model was used to calculate the pooled OR and 95 % CI. A *P* value < 0.05 was regarded as statistically significant. We used χ² and I² tests to evaluate heterogeneity between studies; a χ² test result of *P* value < 0.1 and an I² >50 % indicated significant heterogeneity [15, 16]. Subgroup analysis was performed according to the time of blood sample collection, age, country, presence or absence of infection, stroke severity, onset-to-IVT time, type of HT and type of OR. Moreover, we conducted a sensitivity analysis to assess the contribution of individual studies on the overall effect by excluding one study at a time in chronological order. A funnel chart was used to detect publication bias, Egger’s test was used to evaluate the symmetry of the funnel chart, and a *P* value < 0.05 implied significant publication bias [17]. The trim-and-fill method was used to estimate the effect of publication bias on the results [18]. All statistical analyses were performed by STATA version 16.0 (STATA Corporation, College Station, TX, USA).

## Results

### Study inclusion

**Fig. 1:**
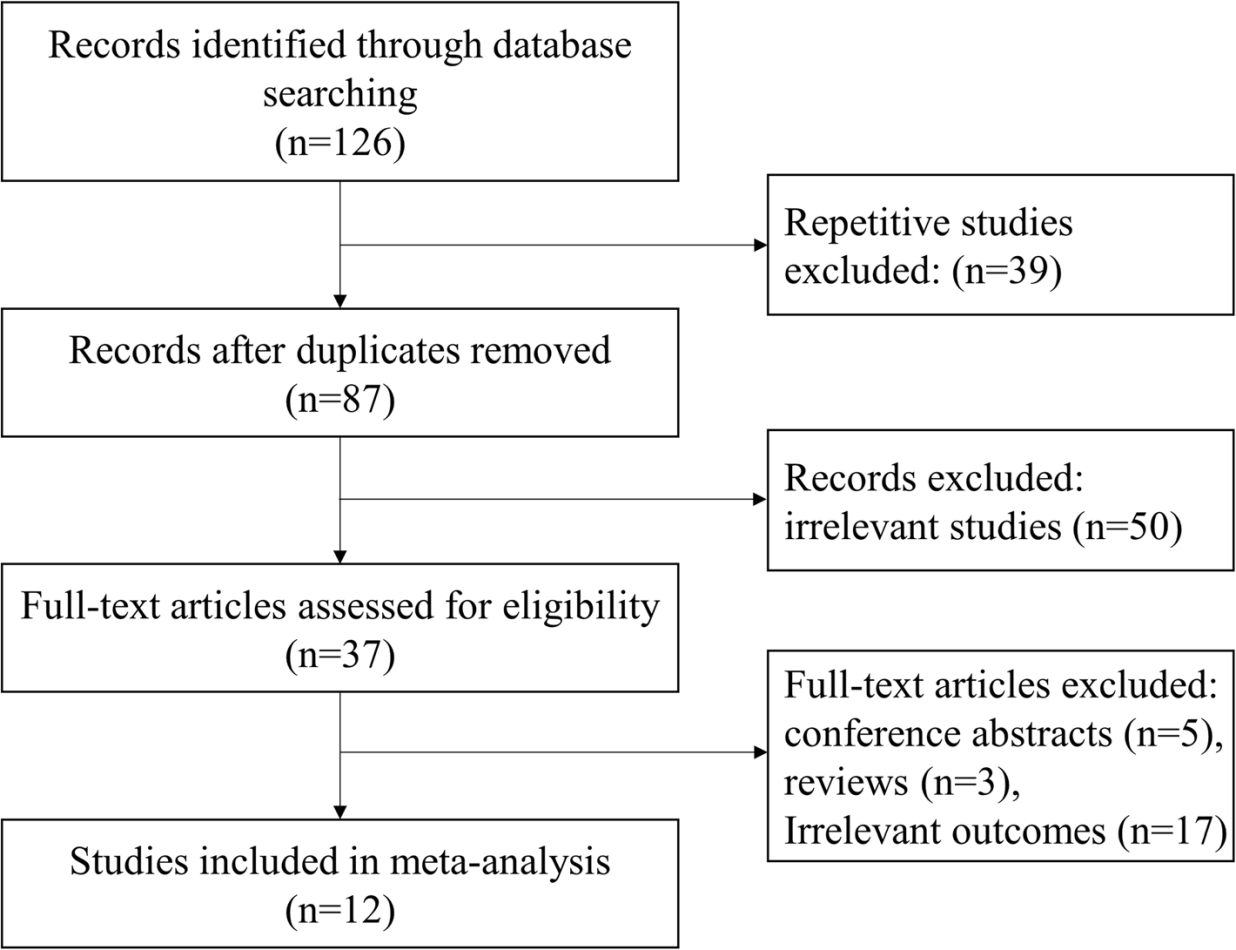
Flow diagram of literatures search and studies selection

The flow chart of the literature searched and included is showed in Fig. 1. After the initial keyword search, 126 articles were included, of which 39 repetitive studies were removed, and 50 irrelevant studies were excluded by reading titles and abstracts. The full text of the remaining 37 articles was read, and 5 abstracts, 3 reviews and 17 irrelevant studies were excluded. Finally, 12 studies were included in this systematic review and meta-analysis [11, 12, 19–28].

### Study characteristics

 A total of 12 studies (*n* = 3641 patients) published between 2015 and 2020 were included in this systematic review and meta-analysis. All 12 studies used standardized intravenous thrombolytic therapy for AIS patients: rt-PA (0.9 mg/kg body weight, maximum 90 mg) was injected intravenously within 4.5 h after the onset of ischemic stroke, with 10 % of the total dose as a bolus and the rest by a 60-minute infusion. Blood samples were collected on admission (*n* = 5) [19, 21, 23, 27, 28], within 24 h after IVT (*n* = 3) [12, 24, 26], or at both times (*n* = 4) [11, 20, 22, 25]. HT (including symptomatic intracerebral hemorrhage and parenchymal hemorrhage), mRS at 3 months, and mortality were reported in 6, 10, and 4 articles, respectively. The best cutoff values of the NLR ranged from 2.2 to 10.59. Since all 12 studies were cohort studies, we used NOS for quality assessment, with a score ranging from 6 to 8 points. The basic characteristics and quality assessment of the 12 included studies are showed in Table 1.

**Table 1:** Basic characteristics and quality evaluation of the included studies

Author	Year	Country	Sample size	Male (%)	Age(year)	Sample time	HT	PFO	Death	Cutoff value	Type of OR	NOS
Maestrini [19]	2015	France and Finland	846	50.8	71(61–80)	On admission	sICH	Yes	Yes	4.8 of sICH	Pooled	7
Guo [12]	2016	China	189	65.1	65.0 ± 10.6	12–18 h after IVT	sICH	NA	NA	10.59 of sICH	Pooled	7
Pagram [20]	2016	Australia	141	NA	74.3 ± 10.7	On admission, 24 h after IVT	NA	Yes	NA	NA	Pooled	6
Malhotra [21]	2018	USA	657	50.7	64.3 ± 14.4	On admission	sICH	Yes	Yes	2.2 of PFO	Crude	6
Shi [22]	2018	China	372	65.1	63.9 ± 13.3	On admission, 24 h after IVT	HT	Yes	Yes	NA	Pooled	8
Guo [24]	2018	China	105	64.8	65.7 ± 9.8	6–8 h after IVT	NA	Yes	NA	5.23 of PFO	Pooled	7
Wang [23]	2018	China	123	65.0	65.2 ± 11.9	On admission	NA	Yes	NA	3.375 of PFO	Pooled	7
Pektezel [25]	2019	Turkey	142	43.7	69 ± 13	On admission, 24 h after IVT	HT	Yes	NA	7.4 of HT, 3.6 of PFO	Pooled	7
Liu [27]	2020	China	285	70.5	62.3 ± 12	On admission	HT	NA	NA	NA	Pooled	7
Ying [11]	2020	China	208	61.5	67.4 ± 12.4	On admission, 24 h after IVT	PH	Yes	NA	NA	Pooled	7
Liu [28]	2020	China	192	71.9	60.8 ± 11.7	On admission	NA	Yes	NA	NA	Pooled	7
Cheng [26]	2020	China	481	61.9	68 (59–76)	24 h after IVT	NA	Yes	Yes	NA	Pooled	7

*HT* hemorrhagic transformation, *PFO* poor functional outcome, *OR* odds ratio, *NOS* Newcastle-Ottawa Scale, *sICH* symptomatic intracranial hemorrhage, *IVT *intravenous thrombolysis, *NA* not available, *PH* parenchymal hemorrhage

### Meta-analysis

#### Hemorrhagic transformation

**Fig. 2:**
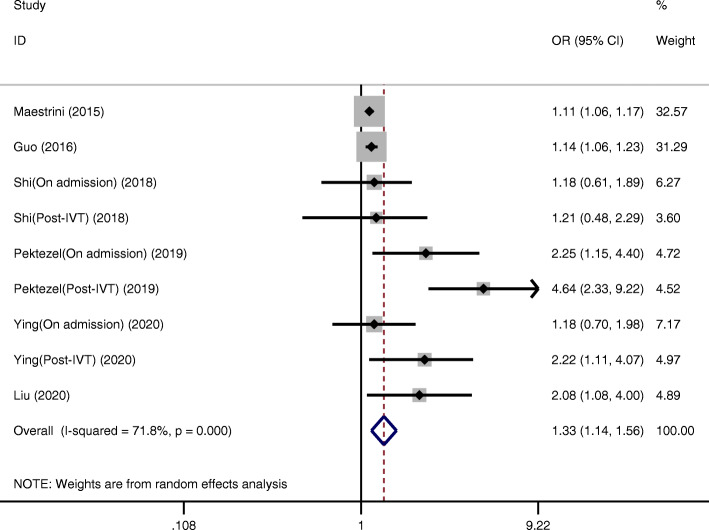
Pooled OR of NLR for HT in AIS patients treated with IVT

Six studies reported the relationship between the NLR and HT after IVT [11, 12, 19, 22, 25, 27]. Higher NLRs were associated with an increased risk of HT (OR = 1.33, 95 % CI = 1.14–1.56, *P* < 0.001). Significant heterogeneity between studies was observed (I²=71.8 %, *P* < 0.001) (Fig. 2).

#### Functional outcome

**Fig. 3:**
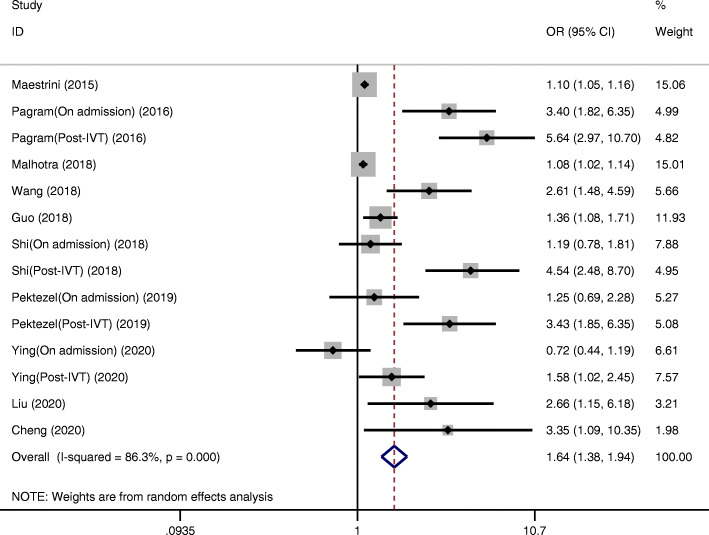
Pooled OR of NLR for functional outcomes in AIS patients treated with IVT

Ten studies reported the association between the NLR and poor 3-month functional outcome (mRS ≥ 3) after IVT [11, 19–26, 28]. Higher NLRs were associated with a higher risk of poor 3-month functional outcome (OR = 1.64, 95 % CI = 1.38–1.94, *P* < 0.001), and significant heterogeneity between studies was found (I²=86.3 %, *P* < 0.001) (Fig. 3).

#### Mortality

**Fig. 4:**
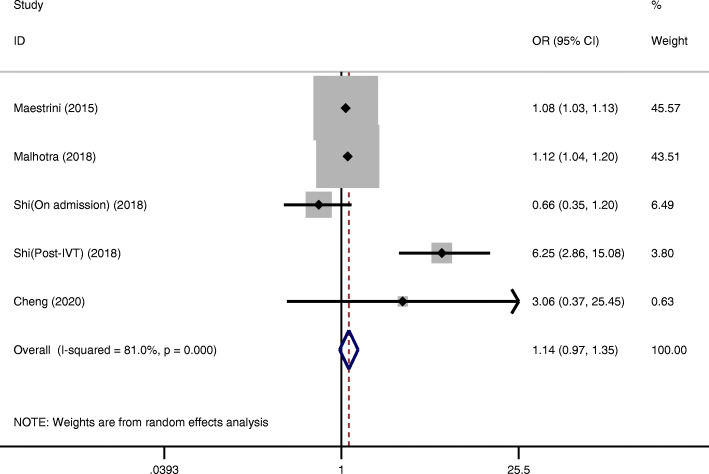
Pooled OR of NLR for mortality in AIS patients treated with IVT

Four studies showed the relationship between the NLR and 3-month mortality [19, 21, 22, 26]. There was no significant association between higher NLRs and a higher risk of 3-month mortality (OR = 1.14, 95 % CI = 0.97–1.35, *P* = 0.120). Moreover, significant heterogeneity between studies was observed (I²=81.0 %, *P* < 0.001) (Fig. 4).

### Subgroup analysis

Subgroup analysis of HT suggested that the NLR at admission rather than the NLR after IVT was associated with an increased risk of HT (OR = 1.33, 95 % CI = 1.01–1.75, *P* = 0.039). A higher risk of HT was observed in the elderly group (OR = 1.32, 95 % CI = 1.11–1.57, *P* = 0.002) and in the studies excluding infection (OR = 1.64, 95 % CI = 1.14–2.35, *P* = 0.008). Interestingly, an onset-to-IVT time less than 3 h was associated with a higher risk of HT (OR = 1.32, 95 % CI = 1.11–1.57, *P* < 0.001). Regardless of the country, stroke severity and the type of HT, the relationship between the NLR and HT remained significant (Table 2).

**Table 2:** Subgroup analyses of the associations between NLR and poor prognosis in AIS patients treated with IVT

Group	HT				3-month poor functional outcome			
	N	OR (95%CI)	*P*	Heterogeneity (I², *P*)	N	OR (95%CI)	*P*	Heterogeneity (I², *P*)
**Sample time**								
On admission	5	1.33 (1.01, 1.75)	0.039	48.7%, 0.100	8	1.22 (1.05, 1.40)	0.007	76.1%, <0.001
Post-IVT	4	1.88 (0.97, 3.63)	0.061	84.7%, <0.001	6	2.78 (1.63, 4.74)	<0.001	84.3%, <0.001
**Age**								
<65	2	1.43 (0.98, 2.08)	0.063	0.0%, 0.393	3	1.83 (1.01, 3.32)	0.045	87.7%, <0.001
≥65	4	1.32 (1.11, 1.57)	0.002	80.0%, <0.001	7	1.87 (1.36, 2.59)	<0.001	87.1%, <0.001
**Country**								
Asian	5	1.68 (1.20, 2.35)	0.003	73.2%, <0.001	7	1.82 (1.31, 2.53)	<0.001	75.5%, <0.001
Non-Asian	1	1.11 (1.06, 1.17)	<0.001	-, -	3	1.37 (1.12, 1.68)	0.002	92.1%, <0.001
**Infection excluded**								
Yes	4	1.64 (1.14, 2.35)	0.008	74.2%, 0.001	6	1.75 (1.24, 2.48)	0.002	75.9%, <0.001
No	2	1.39 (0.77, 2.51)	0.274	71.4%, 0.062	4	1.50 (1.22, 1.85)	<0.001	91.5%, <0.001
**Stroke severity**								
NIHSS<8	2	1.69 (1.11, 2.56)	0.014	30.4%, 0.238	4	1.34 (0.91, 1.96)	0.135	70.7%, 0.008
NIHSS≥8	4	1.25 (1.07, 1.47)	0.006	76.1%, 0.001	6	2.15 (1.50, 3.09)	<0.001	89.9%, <0.001
**Onset-to-IVT time**								
≤3h	4	1.32 (1.11, 1.57)	0.002	80.0%, <0.001	6	1.24 (1.09, 1.42)	0.001	77.6%, <0.001
3-4.5h	2	1.43 (0.98, 2.08)	0.063	0.0%, 0.393	3	2.52 (1.19, 5.30)	0.015	78.0%, 0.003
**Type of HT**								
sICH	2	1.12 (1.07, 1.17)	<0.001	0.0%, 0.558	-	-	-	-
HT or PH	4	1.84 (1.27, 2.66)	0.001	57.0%, 0.030	-	-	-	-
**Type of OR**								
Pooled	6	1.33 (1.14, 1.56)	<0.001	71.8%, <0.001	9	1.96 (1.46, 2.64)	<0.001	86.9%, <0.001
Crude	0	-	-	-	1	1.08 (1.02, 1.14)	0.007	-, -

*NLR* neutrophil to lymphocyte ratio, *HT* hemorrhagic transformation, *AIS* acute ischemic stroke, *IVT* intravenous thrombolysis, *N* number of studies, *OR* odds ratio, *NIHSS* National Institutes of Health Stroke Scale, *sICH* symptomatic intracerebral hemorrhage, *PH* parenchymal hemorrhage

In addition, subgroup analysis of poor 3-month functional outcomes suggested that the blood sample collection time, age, country, onset-to-IVT time, presence or absence of infection and type of OR had no significant influence on the overall results. Higher NLRs were associated with a higher risk of poor functional outcome in the studies with moderate stroke severity (NIHSS ≥ 8) (OR = 2.15, 95 % CI = 1.50–3.09, *P* < 0.001) (Table 2).

### Publication bias and sensitivity analysis

The evidence of publication bias in the studies that reported HT and functional outcomes were detected by Egger’s test (*P* = 0.019 and *P* = 0.001, respectively). After the trim-and-fill test, the pooled ORs were 1.31 (0.99–1.72) and 1.42 (0.86–2.36), respectively (Table S1).

Sensitivity analysis showed that no studies affected the effects of the pooled OR, indicating that the results of this systematic review and meta-analysis were stable (Figure S1, S2).

## Discussion

This systematic review and meta-analysis showed that higher NLRs were associated with a higher risk of HT and poor 3-month functional outcome in AIS patients who received IVT, while higher NLRs were not associated with a higher risk of 3-month mortality. Moreover, this study suggested that the NLR at admission rather than the post-IVT NLR was associated with an increased risk of HT after IVT. These results may be helpful for clinicians to identify the high-risk groups of HT and poor functional outcome after IVT, thereby providing appropriate intervention measures.

Our results suggest that both the NLR at admission and the NLR after IVT were associated with poor functional outcome at 3 months, but the NLR after IVT appeared to have a stronger correlation with poor outcome than the NLR at admission. However, because of the small sample size, future studies are warranted to further determine the best time point of blood sampling for the NLR in predicting outcome in AIS patients receiving IVT. In addition, the cutoff values of the NLR to predict HT obtained from each single paper were 4.8 [19], 7.4 [25], and 10.59 [12] and to predict poor functional outcome were 2.2 [21], 3.375 [23], 3.6 [25], and 5.23 [24], respectively. Of note, although the cutoff values of the NLR varied across the included studies, it is difficult to make pooled analyses to find an optimal threshold of the NLR due to current insufficient data.

The underlying mechanism by which the NLR predicts the clinical outcomes of AIS patients who receive IVT has not been elucidated, and one explanation may be that the NLR combines the inflammatory destruction of neutrophils and the protective effect of lymphocytes. Neutrophils are rapidly recruited to the ischemic area, and microglia are activated after cerebral infarction [5]. Neutrophils release ROS and a variety of inflammatory mediators, chemokines, cytokines, adhesion molecules and proteases to destroy the blood-brain barrier (BBB) to aggravate ischemic injury and brain oedema [6, 29]. Furthermore, neutrophils have been proven to be an important source of matrix metalloproteinase-9 (MMP-9) [30]. MMP-9 was thought to be related to the destruction of the BBB and HT [31]. The rt-PA could not only promote neutrophils to release MMPs but also promote the migration of neutrophils to ischemic tissue through the proteolysis of plasmin and gelatinase [4]. Moreover, rt-PA could induce neutrophil degranulation in vitro [32]. Although reperfusion after IVT is an ideal result, regretfully, it produces more ROS [33]. ROS stimulate microglia and ischemic cells to release inflammatory cytokines and chemokines, resulting in upregulation of adhesion molecules in cerebral vessels, which recruits more neutrophils to the infarct area and contributes to the formation of positive feedback of inflammation after stroke [29, 34]. Lymphocytes play an important role in the protective mechanism of ischemic brain tissue, in which regulatory T cells (Tregs) are important brain protective immunomodulators in ischemic stroke [7]. However, the interaction between the brain and the immune system could lead to systemic immunosuppression in the acute phase of cerebral infarction [8]. Moreover, the stress response led to an increase in the release of glucocorticoids from the hypothalamus-pituitary axis and an increase in the production of catecholamines in the overactivated sympathetic nervous system, resulting in a decrease in the number and activity of lymphocytes [8]. Kim et al. confirmed that a lower lymphocyte count was related to poor outcomes in AIS patients at 3 months [35].

The NLR is a readily available and inexpensive inflammatory biomarker that comprehensively reflects the changes in neutrophils and lymphocytes and provides more information than neutrophils or lymphocytes alone. This systematic review and meta-analysis showed that higher NLRs were associated with a higher risk of HT and poor 3-month functional outcome in AIS patients who received IVT, but considering the limitations of the predictive value of a single biomarker, we suggested that the NLR could be used as a score item of a new prognostic model to predict the clinical outcomes after IVT. In addition, anti-inflammatory therapy may be a potential stroke treatment strategy [36], but previous neutrophil inhibition trials (Enlimomab, LeukArrest and ASTIN) have not achieved the desired results in human trials [37–39]. Exploring the molecular mechanism of adverse outcomes after stroke caused by neutropenia may provide a new therapeutic target for stroke treatment and neuroprotection. Furthermore, with the booming use of mechanical thrombectomy (MT), the relationship between the NLR and outcome after MT is worthy of further research [40].

Significant heterogeneity between studies was found in this systematic review and meta-analysis. Subgroup analyses showed that the heterogeneity of the type of HT, the blood collection time and the severity of stroke were reduced to 48.7 %, 30.4 %, and 0.0 %, respectively, indicating that the blood sample collection time, stroke severity and the type of HT might be the causes of heterogeneity. In addition, significant publication bias was observed. It is necessary for future studies to publish negative results to avoid overestimating the predictive value of the NLR for prognosis in AIS patients who received IVT.

There were still some limitations in this systematic review and meta-analysis. First, some of the studies did not strictly limit infection before admission or after stroke, but the NLR was an important indicator of infection [34], which may have had some impact on the overall conclusion. Second, sICH is a better predictor of clinical outcomes than HT. However, only two studies have reported the relationship between sICH and the prognosis of AIS patients receiving IVT, which deserve further investigation. Third, the cutoff values of the NLR varied in the included studies, which prevented us from determining an optimal threshold. Fourth, all the studies were cohort studies, most of them were retrospective studies and each study had a certain risk of bias.

## Conclusions

Higher NLRs were associated with a higher risk of HT and poor 3-month functional outcome in AIS patients who received IVT. The NLR at admission rather than the post-IVT NLR was an independent risk factor for an increased risk of HT after IVT.

## Supplementary information

**Additional file 1: Table S1.** Publication bias assessment with Egger's test for HT and functional outcome. **Figure S1.** Sensitivity analysis of HT. **Figure S2. **Sensitivity analysis of functional outcome.

## Data Availability

Data generated or analyzed during this study are included in this published article.

## Abbreviations

NLR: Neutrophil to lymphocyte ratio
AIS: Acute ischemic stroke
IVT: Intravenous thrombolysis
OR: Odds ratios
95 % CI: 95 % confidence intervals
HT: Hemorrhagic transformation
mRS: Modified Rankin Scale
rt-PA: Recombinant tissue-plasminogen activator
ROS: Reactive oxygen species
NOS: Newcastle-Ottawa Scale
BBB: Blood-brain barrier
