# Supplementary material for: Prognostic value of the neutrophil-to-lymphocyte ratio in acute ischemic stroke patients treated with intravenous thrombolysis: a systematic review and meta-analysis
Source: BMC Neurol. 2021 May 11;21:191. doi: 10.1186/s12883-021-02222-8 (PMC8111766; doi:10.1186/s12883-021-02222-8)
Supplement: Supplementary file 1 — Additional file 1: Table S1. Publication bias assessment with Egger's test for HT and functional outcome. Figure S1. Sensitivity analysis of HT. Figure S2. Sensitivity analysis of functional outcome. [file 12883_2021_2222_MOESM1_ESM.pdf]

Prognostic value of the neutrophil-to-lymphocyte ratio in acute ischemic stroke patients treated with intravenous thrombolysis: a systematic review and meta-analysis

Chengbing Wang[1],MD, Qian Zhang[2],MD, Mingwei Ji[1],MD, Jing Mang[1*],MD PhD, Zhongxin Xu[1*],MD PhD

[1] Department of Neurology, China-Japan Union Hospital of Jilin University, Changchun, Jilin, China

[2] Department of Cardiology, China-Japan Union Hospital of Jilin University, Changchun, Jilin, China

[*] Both JM and ZXX were the corresponding authors

Additional file 1: Table S1 Publication bias assessment with Egger's test for HT and functional outcome

Publication bias	Egger's *P* value	T&F(Fill) method analysis		Model
		Before	After	
HT	0.019	1.33(1.14-1.56)	1.31(0.99-1.72)	random
Functional outcome	0.001	1.64(1.38-1.94)	1.42(0.86-2.36)	random

Abbreviations: HT: hemorrhagic transformation; Fill: number of studies added by trim and fill method; T&F=result of trimmed and filled analysis, using assumption of random effects.

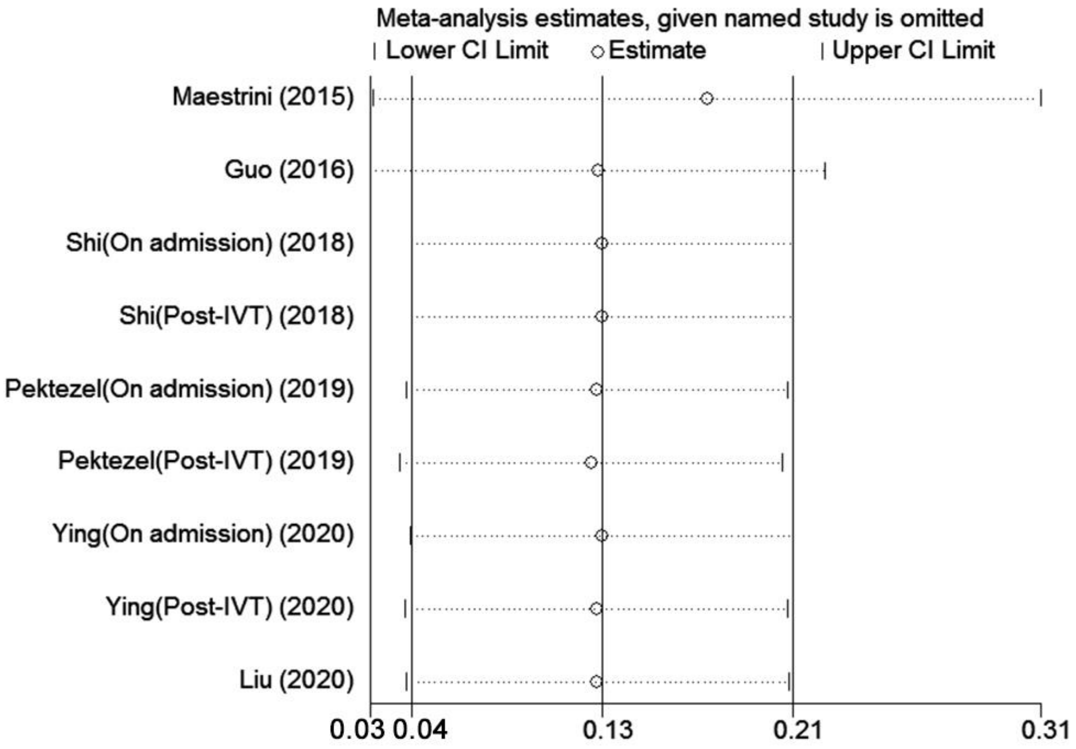

Figure S1 Sensitivity analysis of HT

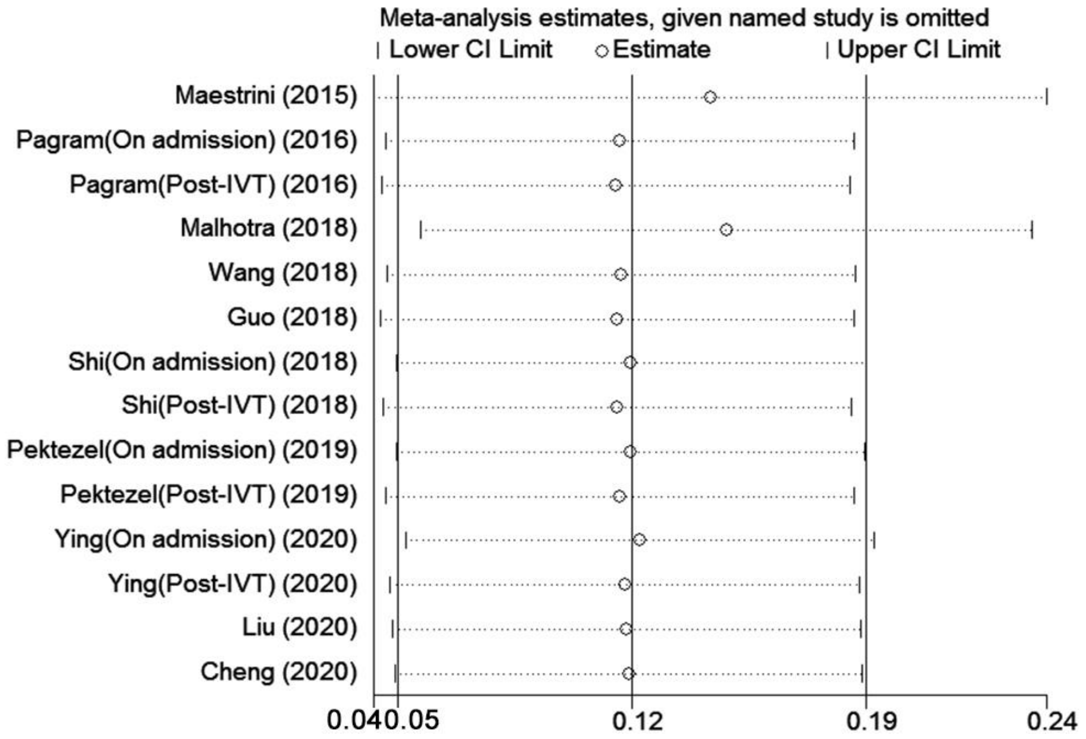

Figure S2 Sensitivity analysis of functional outcome
